# Substances, relationships and the omnipresence of the body: an overview of Ashéninka ethnomedicine (Western Amazonia)

**DOI:** 10.1186/1746-4269-2-49

**Published:** 2006-11-10

**Authors:** Marc Lenaerts

**Affiliations:** 1 Department of International Development, Queen Elizabeth House, University of Oxford, Mansfield Road, Oxford OX1 3TB, UK

## Abstract

Indigenous Amazonian ethnomedicine usually relies on numerous forms of healing, exercised by both specialists and non-specialists. Such is the case among the "Asheninka del Ucayali" (Arawak from the Peru-Brazil border). This paper attempts to elicit the underlying consistencies of their manifold, often contradictory practices and statements.

It draws on ethnographic data gathered between 1997 and 2000, and is essentially based on my own interviews and participant observation. Concerning some specific points these data are also compared with ethnobotanical findings, to highlight significant peculiarities of the Asheninka approach.

The first question is about the nature of a "good medicine". When the Asheninka borrow botanical knowledge from another ethnic group and comment the fact, the contrast between indigenous self-assessments and objective ethnobotanical measurements points out a crucial difference: While the Western approach focuses essentially on chemical effectiveness of the plants themselves, Asheninka people pay much more attention to relational aspects.

The relational dimension also involves the plants themselves, as a sort of person. The point has implications in Asheninka shamanism and herbalism. A shaman does not necessarily need to be a good botanist. His main concern is managing a network of personal relationships involving all kinds of living beings. This network is supposed to be the mainspring of illness – a belief shared by both shamans and ordinary people.

However, most ordinary people have detailed herbal knowledge. In fact, this everyday herbalism amounts to an alternative explanatory model. Such a coexistence of two contrasting explanatory systems is frequent in Amazonia. Among the Asheninka, nevertheless, the underlying hierarchy is clear: the herbal, apparently more materialistic, approach is embedded in the shamanic, plainly relational, model.

## Background

Like many other Amazonian people, the "Ashéninka del Ucayali" and "del Gran Pajonal" have a complex, detailed ethnomedical knowledge. Different forms of healing are performed by shamans, by steam bath specialists and by most ordinary people. It results in a wide range of medicinal means and techniques, with important local and individual variation. Various psychoactive drugs (tobacco, *ayahuasca*, datura...) and countless medicinal plants are used, for steam baths and divination, collective *ayahuasca *ceremonies and individual *ayahuasca *healing sessions, baths of leaves, plant compresses, plant juices used as eye drops, tobacco and other specific plants used for juice spit, blowing of tobacco smoke, little pieces of thorn or charcoal extracted from the patient's body...

Together or separately, similar practices are widespread throughout Amazonia, but rather than comparative work, my concern here is with the internal consistency of such a variety, from an Ashéninka point of view.

At the moment, various researchers offer very stimulating insights into the healing systems and medical concepts of the closely related Matsigenka [1-3] and other indigenous neighbours [4-6]. By contrast, the Ashéninka or Asháninka ethnomedicine has never been addressed addressed as a whole. Even partial studies on this topic are very few and focus exclusively on Asháninka shamanism [7,8] or pharmacology [9].

### Ethnobotany and indigenous categories

These studies actually fit in with two traditional focuses of interest in Amazonian ethnomedicine. The first one addresses the shamans, their social role, symbolic thought and publicly ritualized action, i.e. the most dramatic aspects of healing [[10-15], among many others]. In Western Amazonia it often entails a particular attention paid to the use and diffusion of *ayahuasca*, the main shamanic hallucinogen in the region [16-20]. The second one focuses on ethnomedicinal plants and pharmaceutical properties, especially the mentioned *ayahuasca*, *Banisteriopsis caapi *(Spruce) Norton, Malpighiaceae [21-23], and other outstanding species [24-26].

My own concern is rather different. Facing the great variety of everyday healing (which includes much more than just shamanic practices) I aim to determine its underlying conceptual framework, according to the Ashéninka categories of thought. The whole paper defends the proposition that a prior condition for any medical anthropology in the Ashéninka case is a thorough examination of Ashéninka epistemology.

The first steps of the analysis are based on findings of our interdisciplinary work. They substantiate that the Ashéninka approach is inconsistent with Western categories, and calls for another analytic framework in the next steps. This is the reason why I progressively abandon the references to and comparison with ethnobotanical data, after applying them to clarify the contrast between both approaches.

The ontological backgrounds are different: There are indigenous conceptions about bodies and "souls", material substances and interpersonal relationships, but these categories do not have exactly the Western sense, and most of all they are interconnected in a distinct, non-Western way. I argue that the point is critical to both sides of Ashéninka ethnomedicine, namely shamanism and herbalism, and to their balance and interaction.

Herbal knowledge is widespread among most of the Ashéninka people, but paradoxically it is also poorly valued in everyday comments. A typical illustration is the following example. I had broken my leg, and the Ashéninka taught me to use a plant with amazing anti-inflammatory effect (I give fuller particulars in the section "results"). I repeatedly expressed my grateful admiration for such a "good medicine", but the Ashéninka's comments on it were greatly contrasting. Scornfully, they answered: "*Everybody knows that plant, even the children*".

Which is the real status of such herbal knowledge then? Does it belong to a specialized, almost separate field of Ashéninka ethnomedicine, as it may occur elsewhere in Amazonia, among the Warao [27] or many Panoan groups [28,29] for instance? Is there a sharing out between a spirit dimension of healing, reserved to shamanism, and a bodily one, reserved to the layman's herbalism, as assumed in the common sense opinion?

I do not think so. I argue that Ashéninka herbalist approach rather is a second-class, symptomatic treatment of the *same *health issues, and is viewed as a mere alternative *expression *of the same processes, involving primarily the highly transformable *bodies *of human people and other living beings. To substantiate this point, the overall context will be detailed first, and roundabouts through shamanic practices and their ontological background will be necessary: Ashéninka ethnomedicine is based on values and categories of thought quite different from ours, and is to be addressed as a whole.

### Ethnological background

The lack of bibliographical references on Ashéninka/Asháninka ethnomedicine is a little surprising, considering the population size. The Ashéninka and Asháninka together amounted to more than 51,000 people in 1993 [30]. They belong to the Arawakan ethno-linguistic group, and dwell in the Peruvian "Selva Central", with some territorial extensions going across the Brazilian border [31]. Like many other Amazonian peoples, their economy is based on slash-and-burn agriculture, hunting, fishing and gathering – and nowadays, some additional commercial crops and activities (coffee, red beans, timber...). I personally worked with two north-eastern sub-groups, known as "Ashéninka del Ucayali" (above 3,500) and "Ashéninka del Gran Pajonal" (almost 4,000), in the Peruvian Department of Ucayali and the Brazilian State of Acre.

Peruvian people usually deem the Ashéninka to be very rebellious. Their history is strewn with many armed uprisings [32-35], and for a long time they fought quite successfully against repeated colonization attempts by the Peruvian state, by the Spanish conquistadors and missionaries, and still before, by the Inca empire [36]. Even in more peaceful situations, there is clear evidence of their deep desire for autonomy and strong ethnic pride [37,38].

**Figure 1:**
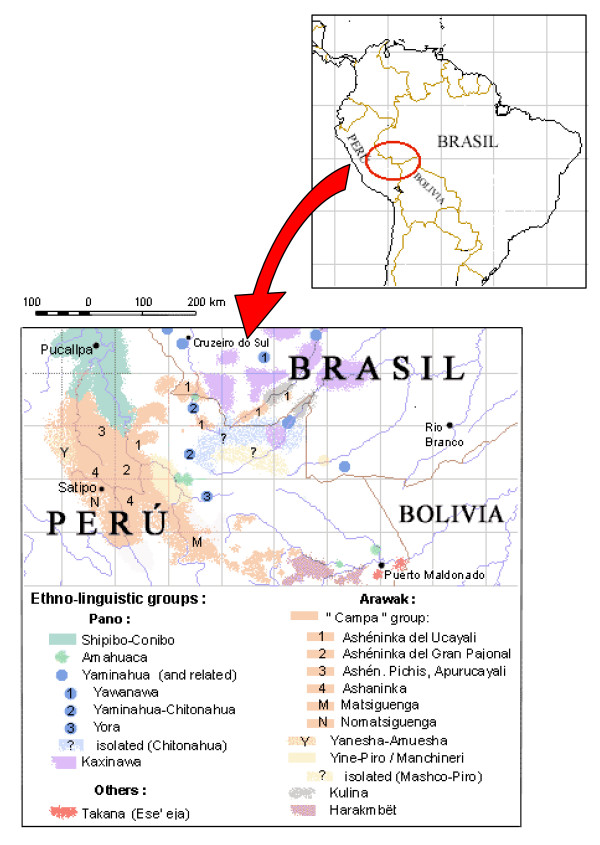
Ethno-linguistic map (Ucayali and Madre de Dios, Peru; Acre, Brazil). Sources: Chirif & Mora, AIDESEP, Centro Eori, ILV-SIL (Peru), CEDI, Governo do Estado do Acre (Brazil), and personal data.

Some of their neighbours will be mentioned in this paper. Besides the Ashéninka and Asháninka, the Sub-Andean Arawak also include the already mentioned Matsigenka to the South-East, the Amuesha-Yanesha to the West, and the rather distinct Yine-Piro (who are ancient enemies) to the East. Their traditional territories formed a solid block (see the map in Figure 1).

Beyond, most of the indigenous neighbours belong to the quite different Panoan ethno-linguistic group. All of them were enemies of the Ashéninka, Asháninka and Matsigenka. They present nevertheless contrasting cultural features and history. On the one hand, the Conibo and Shipibo also form a large indigenous group (more than 20,000 people in 1993 – see [30]), settled for centuries on the great river banks [39]. Most of them still live along the middle Ucayali, the main artery of the region, and some of its tributaries, often near Peruvian settlements and cities. On the other hand, there is a set of small ethnic groups, loosely related to each other. Traditionally, they lived scattered in the headwaters regions. Those who will be mentioned in this paper were successively drawn to sedentary settlements in rather recent times, after an "isolation" of many ages in the forest: Permanent contact with the Yaminahua (around 400 people in 1998) occurred in the 60's [40-42], and with the Yora (around 230 people in 1999) in the 80's [1,43]; the sedentary settlement of the Chitonahua (around 150 people?) began ten years ago and is still in process [1,40,42].

## Methods

The present case study draws on ethnographic research carried out between 1997 and 2000. It is essentially grounded on my own data and findings as an anthropologist. I stayed among the Ashéninka in seven villages from Brazil and Peru, with special attention paid to knowledge variation and to healing everyday practices and ceremonies.

The basic methods were participant observation and open-ended interviews. In-depth semi-structured interviews were not carried out from the outset, in order to limit self-induced answers and bias of the indigenous categories. Rather, they were used some later to gather additional data and crosscheck previous information and provisional interpretations. I worked in this way with most members of three villages (Alto Bonito, 43 people; Dulce Gloria, around 250 people; Nueva Victoria, 95 people), and with many members of the others. Interviewees were shamans as well as ordinary people reputed to be either skilled or inexpert in ethnomedical issues. The large variety of informants and direct observations demonstrated the great variability of healing practices among the Ashéninka.

Nevertheless, my personal research formed part of a wider programme granted by the European Commission and entitled "*TSEMIM*" (*Transmission et Transformation des Savoirs sur l'Environnement en Milieux Indigènes et Métis*). Concerning some specific points, this paper also draws on findings of the colleagues involved in this *TSEMIM *research, in order to enlighten some significant peculiarities of the Ashéninka approach. Since this wider research programme forms part of the background, I give some particulars about its objectives and methods.

The purpose was to analyse the dynamics of change and transfer of environmental knowledge among six neighbouring indigenous peoples, first within each ethnic group (focusing on the chances of trans-generational transmission), and further between all of them (focusing on interethnic exchange and influences). These ethnic groups were the Shipibo-Conibo, Amahuaca, Yaminahua-Chitonahua, Yora and Yawanawá (all of them Panoan), and the Ashéninka (Arawakan).

The interdisciplinary teams included anthropologists, botanists and ethnobotanists from Brazil, Peru, France and Belgium. I use and quote in this paper the findings and personal communications of the anthropologists Frédérique Rama Leclerc (Shipibo-Conibo), Rodolfo Tello Abanto (Yora), Miguel Carid Naveira and Laura Pérez Gil (Yaminahua-Chitonahua, Yawanawá), and botanists Niels Valencia Chacón, Joaquina Albán Castillo, Betty Millán Salazar, Eduardo Salas Zuluaga, Severo Baldeón, Rosa Bueno Cuadra y Carmen Martínez.

The anthropologists worked in parallel, each of them with a specific ethnic group. They began first with a long term, preparatory fieldwork. Later, they were joined each in turn by relatively steady teams of botanists and ethnobotanists from the *Museo de Historia Natural de Lima*, who carried out a one-month systematic survey among each ethnic group. This botanical fieldwork was systematically attended by the local anthropologist.

Due to the target of the research, i.e. knowledge variation, (ethno)botanists as well as anthropologists had to choose informants deliberately varying in age, gender and knowledge reputation, and the enunciation context of all indigenous answer and information (either spontaneous or not) was carefully recorded in a suited field sheet, and later in the database. Among the Ashéninka, the botanical survey for instance was carried out in this way in three villages (Dulce Gloria, Nueva Victoria and Aerija), with a total of 42 informants. Their participation to the identification work in the forest plots was voluntary, but constant attention was paid to diversify the small groups (2–6 persons) who were successively involved in the field trips.

Regarding the botanical collection, the basic method was Gentry's lineal transects [44]. Priority was given to an intensive inventory to take into account vines as well as trees. That was the reason why we opted for a low minimum diameter at breast height (DBH) rather than for a large number of plots [45]. These plots were transects of 50 × 4 m., located in primary or late secondary forest. The sites were chosen by the botanists within a 90-min walk of the settlements, which is a very usual distance for indigenous gathering, but outside of the places previously best known by the informants, in order to test their knowledge on a random basis. All plant individuals with a DBH of 2.5 cm and above were tagged and identified as far as possible (unfortunately, part of them only at genus level; those were not taken into account in the further full comparative work with the database). Indigenous informants were asked about the name, use and detailed forms of use of all these plants.

In addition, a subsidiary inventory also addressed plants of smaller DBH, but of greatest interest for ethnobotanical analysis and intra- or interethnic comparison. It included juvenile trees as well as shrubs, vines, herbs, epiphytes and even non-vascular plants, pointed out by the ethnobotanist, the anthropologist or the indigenous informants themselves, in the transects, in the fallows and during the field trips.

A special attention was also paid to the protection of indigenous intellectual property rights. Since our research was not expected to entail any direct industrial or commercial application, we did not address these rights focusing on benefit sharing, as very correctly suggested by the Declaration of Belem or D. Posey, for instance [46,47], but rather in a defensive way. All scientists and institutional partners committed themselves to keeping all sensitive data secret. In addition, after our own comparative work, a random link disconnected botanical identifications and indigenous uses in the central structure of the database (even in the copies provided to the European Commission), which makes it completely useless for pharmaceutical, parapharmaceutical or biotechnological purposes. Prior informed consent of indigenous communities and political organizations was obtained both in Brazil and Peru, but the Brazilian authorities eventually did not allow ethnobotanical collection. The research was thus pursued on the only Peruvian side, without the expected ethnobotanical data on the Yawanawá and Brazilian Ashéninka. The voucher specimens (all of them collected in Peru) were stored at the *Museo de Historia Natural de Lima*.

Botanical, anthropological and consolidated reports of this research programme were published some years ago [48]. I was in charge of the synthesis of the results. I assume entirely the possible errors of interpretation it could entail, both in that former publication and in the present paper.

## Results and discussion

### 1. What is a "good medicine"?

What actually is a "good medicine"? At first sight, the answer seems to be obvious. A medicine must be efficient at curing injuries and sicknesses, and according to Western common sense, such efficiency relies essentially on chemical effects. The potential discovery of new active molecules is probably the main reason for the widespread interest in tropical ethnomedicine [49-52]. In this sense, local survival of extensive traditional knowledge of medicinal plants appears as a guarantee of success: Some of these plants at least are likely to be really (i.e. chemically) active... If one does not feel particularly interested in indigenous categories of thought, the next most important step is just the scientific analysis of the plant components, in order to test their "real" efficacy.

Nevertheless, the indigenous approach may be quite different. All Ashéninka I met know a very large range of medicinal plants [[53,54], and personal observation]. Given the strong ethnic pride I mentioned before, they should be expected to have a high opinion of this knowledge. In this respect, however, they have always the same curious comment: Either spontaneously or prompted, they inevitably assert that the people who really know about this matter are not themselves, but their Shipibo neighbours, who live closer to the river Ucayali, the main artery of the region, and generally closer to the Peruvian settlements and cities.

#### "Who really know about plants are our neighbours"

This acknowledgment of Shipibo superiority is actually much more than a simple comment. It entails direct borrowing of ethnobotanical knowledge as well. The qualitative comparison between ethnobotanical records among both ethnic groups substantiates that Shipibo knowledge is massively adopted by some Ashéninka individuals [[53-56], and comparative work with the *TSEMIM *database]. These individuals are a little distinct from the others, even in their personal history. Usually they have (or have had) more extensive contacts with the outside world, and could be called the most "cross-cultural" Ashéninka. At the same time their personal history gave them the opportunity to come into contact with Shipibo healers. Actually they adopted many Shipibo characteristic plants and forms of use, and (perhaps more significantly) also many aspects of the Shipibo's peculiar style of management [personal communication of F. Leclerc, [57]]: Around their house, these "cross-cultural" individuals grow various "master-plants", considered as a sort of panacea, they engage in new, more individualistic, forms of shamanism, they openly talk about witchcraft plants, and so forth – which the other Ashéninka never do. Moreover, this knowledge borrowed from the Shipibo does not come in addition to the Ashéninka "traditional" one, but partially substitutes it [58].

At first sight, it seems to be absolutely normal: The most travelled and probably open-minded Ashéninka are giving up part of their own ethnomedicinal knowledge and learning the Shipibo one – why not, if the latter is better?

Nevertheless, the comparison with the results of the botanical and ethnobotanical surveys proved to be rather surprising. Contrary to all expectations, the Shipibo informants did not identify a wider range of medicinal plants than the Ashéninka. Among many other kinds of data, the botanists had recorded how many plant samples had a specific indigenous name, and how many had a specific use. These kinds of rates are commonly used as a first assessment about the overall level of local knowledge [45,59]. The amazing point was that regarding *all *these rates, the Ashéninka informants had reached much better results than the Shipibo ones, as substantiated by Tables 1, 2 and 3.

**Table 1:** Ashéninka and Shipibo general ethnobotanical knowledge

**Ethnic group**	**reference samples**	**reported name and/or use(s)**	**reported specific use(s)**	**medicinal use(s)**^**1**^
**Ashéninka**	601	98.7 %	97.0 %	78.0 %
**Shipibo**	258	87.6 %	86.1 %	71.5 %

(source: *TSEMIM *database)

^1 ^In the indigenous sense, that is to say including also a wide set of uses and effects that the Western approaches usually deem to be "magic" or "irrational", but that indigenous people do not really distinguish from the rest of medicine: shamanic hallucinogens, hunting magic, protection against harmful spirits, enhancing of children skills, seduction medicines, and so forth – see Table 3.

**Table 2:** Distribution of indigenous uses (general)

**Ethnic group**/Local settlement	Total used species samples	food	handicraft	body care	market	house building	fire wood	hunting – fishing		medicine and related
									
								baits & fishing poisons	magics	
**Amahuaca**										
Boca Pariamanu	317	6.6 %	4.4 %	0.9 %	0.9 %	2.8 %	1.3 %	0.3 %	1.3 %	51.7 %
**Ashéninka**										
Aerija	373	7.8 %	2.4 %	6.2 %	3.5 %	6.2 %	2.7 %	0.5 %	2.7 %	76.7 %
Dulce Gloria	345	7 %	3.2 %	2 %	2 %	3.2 %	0.3 %	0.9 %	4.3 %	82 %
Nueva Victoria	316	3.2 %	4.1 %	1.6 %	0 %	1.6 %	0.3 %	1.3 %	4.1 %	88.6 %
**Shipibo**										
San Francisco	211	2.4 %	6.6 %	3.8 %	0 %	1.4 %	0 %	2.4 %	2.4 %	86.7 %
Santa Rosa	249	2.4 %	8.4 %	2.4 %	0.4 %	2 %	0.4 %	0.4 %	3.6 %	80.3 %
**Yaminahua**										
Raya	546	2.4 %	1.3 %	0.2 %	0.5 %	2.4 %	0.2 %	0.5 %	2.4 %	88.1 %
**Yora**										
Santa Rosa de Serjali	844	14.5 %	4.7 %	2.4 %	0.5 %	4.7 %	1.3 %	0.5 %	0.9 %	60.7 %

Source: *TSEMIM *database

**Table 3:** Distribution of indigenous uses (medicine)

**Ethnic group**	Total used species	biomedical sense	cure of *cutipado *& susto ^1^	protection and propitiation	hunting-fishing magics	children socialization	shamanism	indigenous sense (total)
**Amahuaca**								
Boca Pariamanu	317	45.1 %	3.8 %	2.5 %	1.3 %	0.9 %	4.1 %	51.7 %
**Ashéninka**								
Aerija	373	66 %	6.2 %	2.4 %	2.7 %	3.8 %	1.9 %	76.7 %
Dulce Gloria	345	58 %	9.9 %	7.5 %	4.3 %	2.9 %	3.8 %	82 %
Nueva Victoria	316	61.1 %	15.5 %	7.6 %	4.1 %	0.9 %	2.8 %	88.6 %
**Shipibo**								
San Francisco	211	62.6 %	7.1 %	20.9 %	2.4 %	1.9 %	8.5 %	86.7 %
Santa Rosa	249	55 %	3.2 %	23.7 %	3.6 %	1.2 %	0.4 %	80.3 %
**Yaminahua**								
Raya	546	74.5 %	1.5 %	5.7 %	2.4 %	0.7 %	4.6 %	88.1 %
**Yora**								
Santa Rosa de Serjali	844	55.9 %	1.1 %	3.1 %	0.9 %	0.9 %	1.2 %	60.7 %

Source: *TSEMIM *database

^1 ^harmful influence of spirits, animals, etc.

Actually, it could also be argued that the Shipibo medicinal plants should be more efficient, but there is absolutely no evidence of such a qualitative superiority. I am absolutely conscious that I cannot prove my statement in a strict scientific way. We did not test the pharmaceutical efficacy of any medicinal plant we discovered or registered. It was outside the scope of our research, and besides we committed ourselves to the total protection of the indigenous intellectual property rights (see details in section Methods). However, in my opinion some particulars could be considered as significant indications.

On the one hand, coincidences in fieldwork gave us the opportunity to experiment with Ashéninka's medicinal plants. I can testify that some of them are quite efficient, in a strict biomedical sense. My first example is about a disinfectant/cicatrizing compress (please forgive me if I keep the botanical identifications confidential: that was our personal commitment with indigenous informants and organizations). It was used on a thumb tip cut to the bone. After exactly seven days, the cicatrisation was perfectly formed, without any infection. My second example is an anti-inflammatory leaf. I had broken my fibula, but I thought it was just a sprain. I had to walk as I could to deal with everyday needs and my ankle was terribly swollen. The effect of the anti-inflammatory leaves was amazing. Directly under the compress, the swelling used to go down spectacularly after a couple of hours. After two weeks, I was able to walk up and down the hills, and to go back to the closest landing strip.

On the other hand, the Shipibo herbal medicines have been well-known for many years. They were widespread beyond the ethnic boundaries due to training programmes [60] and popular handbooks [[61] – see also [62-64]]. In the Ucayali region, they belong now to the public domain. Some of them are presumably efficient too, but as far as I know, none is particularly famous for its outstanding, immediate efficiency in a strict biomedical sense. The best of Shipibo medical skills lie probably elsewhere.

In my eyes, there is thus no doubt that the Ashéninka herbal medicines are likely to be as efficient as any Shipibo equivalent. If so, we are faced with a strange paradox. According to most common Western criteria, the Ashéninka ethnomedicinal knowledge is really outstanding, but the proud, self-esteeming, Ashéninka themselves did not agree at all. From their own point of view, this knowledge was so underestimated that some of them preferred to borrow from foreigners – who actually do not seem to be more knowledgeable... It looks like an absurdity. That was our first surprise, but in fact we were struck by a second one: In the Ucayali region, this kind of situation was anything but an exception.

#### Regional chains of interethnic borrowing

Besides the Ashéninka, the *TSEMIM *research programme addressed five other indigenous groups in the same way. The final comparative work on all anthropological and botanical data highlighted another finding: Despite its paradoxical nature, the borrowing relationship between Shipibo and Ashéninka actually corresponds to a rather frequent pattern. I do not mean that it could thereby be used as a general model for inter-indigenous relationship in Amazonia: Even in our work region, a few counter-examples could be found. But the repetition of the same pattern is conspicuous enough to warrant full attention.

The same schema was repeated several times, forming chains of interethnic borrowing. Just as the Ashéninka borrow from the Shipibo, others, namely the Yaminahua, borrow from the Ashéninka, while a fourth group, the Chitonahua, borrows from the Yaminahua, and a fifth one, the Yora, from another sub-group of Yaminahua.

All these cases present the same characteristics. Firstly, the adoption of new ethnomedicinal knowledge does not seem to rely primarily on pragmatic or pharmaceutical substantiation. Secondly, it is a large scale process. Borrowing includes crucial species and entails important changes in both the empirical management of the plants and the social management of knowledge. According to Miguel Carid Naveira's and Laura Pérez Gil's findings, the Yaminahua for instance formerly used to gather wild plants in the forest: It was a mostly male activity, and the only restriction to access these resources rested in individual levels of ethnobotanical knowledge. Currently, women's involvement has become more important: They have obtained varieties of cultivated "piri-piri" (*Cyperus *sp., Cyperaceae) from the Ashéninka, but have also learned how to preserve the related knowledge as a personal *secret *– and they are now managing *wild *medicinal plants in the same way. It is a complete reversal of former customs [42].

Moreover, the orientation of borrowing chains is very consistent – and that is probably the crucial point. Despite traces of a formerly much more complex situation [37], the process is restricted nowadays to a single direction: The new knowledge always comes from the indigenous neighbouring group who is closer to the Peruvian urban world.

In this respect, the interethnic position of the respective groups is quite significant. As mentioned before, the Shipibo are living very close to the urban people (their main village is actually a suburb of Pucallpa, the second big city of Peruvian Amazon), and they are very skilful in trading with non indigenous people [65,66], in promoting their shamanic skills and in negotiating with Peruvian institutions. In a city like Pucallpa, numerous *mestizos *and foreign people consult Shipibo shamans. They visit them for relational, emotional and psychosomatic problems (envy, personal success, love problems, illnesses including behaviour disturbance like "*susto*" and "*cutipado*"...) much more than for herbal treatment (D. Lacaze and P. Deshayes, personal communication). Besides their skilful management of handicraft and shamanic cures oriented to *mestizos *and foreigners, the Shipibo also obtain many little jobs in the civil service, as indigenous teachers for instance. Statistics were not available, but empirical observation reveals a conspicuous overrepresentation.

The Ashéninka, despite centuries-old contacts, tend to be much more reserved. The jobs they obtain in the civil service tend to be restricted to a very local level. The three other ethnic groups are ancient isolated people, successively drawn to sedentary settlements: the Yaminawa were in the 60's, the Yora in the 80's, and the first Chitonahua came out from the forest in the last 10 years – part of them remaining still isolated [42,67].

What does borrowing mean, then? Obviously, here the mixed-blood society is a crucial focus of attraction, although it is in a complex and oblique feature. The borrowing process partially means going towards the Peruvian world. But at the same time, new knowledge comes from indigenous neighbours. Moreover, all of the new borrowed items are emblematic of "indianness": They concern ayahuasca (a shamanic psychoactive mixture), manioc beer, shamanism, steam baths, or the already mentioned piri-piri... The result is a closer proximity to urban people, though keeping very clear indigenous features.

What is at stake here is thus a question of collective identity, that is to say the construction of a defined place in a shared interethnic system, much more than something *we *should call therapeutic efficacy. For us, such a thought reveals a sort of "confusion" between collective identity, interethnic relationship, and medical efficiency. However, we have absolutely no reason to suspect the sincerity of indigenous belief. Health is a major concern for them, and what is borrowed from the neighbours has to be used in the flesh. When comparing respective medicinal skills, the "inconsistent" hierarchy indigenous people build is nothing but their own way to assess what *they *deem to be *actual *therapeutic efficacy.

I repeat that we cannot really compare biomedical efficiency here, given the lack of chemical and pharmaceutical evidences. I just want to stress the contrast between indigenous and Western thought processes. For the Ashéninka (and presumably the other peoples I mentioned too), the first concern is with respective positions in the interethnic landscape. Presumptions of plant efficiency are induced from this prior criterion. Empirical confirmations may occur later, but just as a last, optional step. For Western people and scientists, the first concern is with the plants. The extent and diversity of local knowledge (rather than its overall fame) may be taken as a promising indication, but nothing is achieved before chemical analyses. The former is based on relationships, the latter on material substances.

Let me emphasize that the indigenous approach is quite different from inability to take the conclusive empirical steps, or from attention simply paid to some additional dimensions of medicine. Rather, it reveals two contrasting conceptions and managements of medical knowledge.

For biomedicine and ethnobotanical science, the healing power of a medicinal plant belongs to the world "out there". Both the plants and the related knowledge are managed as mere objects. They may be bought, borrowed and transferred independently of the people who knew and used them first. In order to be scientifically tested and prove their therapeutic efficacy, they *must *be "isolated" (the active substance from the plant, and the plant itself from its local context). Knowledge is a matter of neutral, universal verification.

By contrast, in the Ashéninka approach, neither the plant nor the knowledge may be separated from people and relational contexts. Their healing power is understood to come precisely from their respective position in an overall network that includes plants as well as human beings. The Ashéninka trust the Shipibo medicinal plants primarily because they are related with Shipibo people, and because Sipibo people themselves are related with urban people (P. Gow argues for the same kind of motivation concerning the diffusion of ayahuasca throughout Western Amazonia [68]). Pharmaceutical or chemical effects are taken into account later on, as a second-class criterion. Knowledge is a matter of ties between plants and persons keeping in touch.

### 2. Who is a "good healer"?

The analysis of borrowing processes led us to a crucial criterion: the opposition between substances and relationships. Priorities are quite different for Western and Ashéninka peoples. The contrast could also be expressed in other words. For biomedicine, a good healer is somebody who suitably uses good medicines. For an Ashéninka, the reverse makes much more sense: A good medicine is something used by a good healer.

The latter should be a mere tautology if it were not underlain by a complex conception of healer's agency. It is worth noting that placing stress on the relational background of medicinal resources and knowledge is not so unusual. Even in our own societies, some people assess the therapeutic value of exotic or native treatments according, primarily, to their mystical/geographical or ecological/natural source. But it is a peripheral claim, usually referred to poorly known "alternative" traditions. By contrast, the Ashéninka relational approach is grounded in a dynamic and quite consistent system of thought and practice.

#### The great shaman who knew very few plants

I question the point starting from an occasional case, a (great) shaman who hardly could identify wild medicinal plants. The example might be a little surprising and is not to be generalized, but it does not seem to be so exceptional. Most of all, it enlightens the peculiarity of Ashéninka priorities.

In the border region of the river Yurua (Juruá in Brazil), Shoéshi is known as a very great shaman. His reputation extends far beyond his little village, Nueva Victoria, on the Peruvian upper reaches of the river.

With the botanists Severo Baldeón and Eduardo Salas Zuluaga (both from the *Museo de Historia Natural *of Lima), Shoéshi did not take part in the ordinary informants teams, but we managed to take him along on a couple of special ethnobotanical walks. As usual, a few other people were coming along (we did not want to impede it, given our special interest paid to enunciation contexts and informants' interaction), but very quickly we realized that something was going wrong. Whenever the plant identification was likely to be more difficult or dubious, Shoéshi trailed behind, while all the others (namely his wife, two of his nephews, one of them BS and the other ZS...) rushed to answer first. The ethnobotanist obviously managed to prevent such an intromission and forced answers from Shoéshi himself. The experiment provided us with substantial evidence that the greatest shaman of the Yurua border also had one of the lowest identification scores we had measured: no more than 70 or 75 % of the average adult's results [[53] and *TSEMIM *database].

However, Shoéshi is not a charlatan, but a real spiritual leader and quite an effective healer. Ill people pay him constant visits. Some of them are coming from nearby settlements, but others are from Brazil, which means travelling through the forest for a couple of weeks and staying in Nueva Victoria for several months. His healing reputation even cuts across ethnic boundaries: I alluded before to the Yaminahua women who visit him to learn about his steam baths techniques. Actually, the question is not about Shoéshi's efficacy itself. It is a more fundamental one: How could somebody be so great a plant healer, with so poor botanical ability?

A first and very circumstantial point is that Shoéshi does not have good eyesight, due to congenital trouble. Of course his relatives are well aware of that weakness. Theirconstant willingness to answer instead of him, all along the two identification walks, is quite understandable: Such a crude systematic test could not but humiliate Shoéshi. Moreover, it was still more unfair than it appears at first sight, because of the kind of knowledge this humiliation was paid to: In fact, from the indigenous standpoint it is nothing but a very superficial one.

Despite a frequent prejudice, knowing a large range of medicinal plants is not a shaman's particular skill. In some ethnic groups, such knowledge is rather equally shared by all men and women, as it is among the Ashéninka or the Ka'apor [69]. In others, like among the Yagua [10], the Warao [27] and many Panoan groups [28,29]; [42,43], it is a matter for specialists. These specialists are often distinguished by a specific term, but their knowledge is viewed as an incomplete one, a sort of second-class skill. The herbalists try to intervene first, and if they fail, ill people have to visit the shaman.

#### Herbalism and shamanism

Shamans are deemed to have a superior knowledge, since they are able to heal illnesses that ordinary people or herbalists cannot. But their knowledge is often very elusive, and actually the more encyclopaedic, use-oriented ethnobotany of indigenous herbalists or ordinary people seems to be closer to our own system.

In fact, I suspect that this apparent closeness arises merely from a working misunderstanding (in Marshall Sahlins' sense, [70]), but after all it does work, so that there are few questions about it. However, the underlying inconsistency is rather conspicuous. In many ethnobotanical surveys, for instance, the categories of uses "magic" and "medicine" are clearly contrasted [69]. Other authors have taken a more nuanced approach [71,1,3]. As Western people, nevertheless, we cannot help establishing a clear distinction between a plant that protects you from bad spirits, or brings you luck with hunting, and another that cures you of fever; or between a plant that protects you against a future snake bite and another that stops the effects of an actual one.

There is clear evidence of our common difficulty in dealing with indigenous empirical categories of thought. However ingenuous it may be, our most spontaneous reaction is to refer all "magic" or "irrational" uses to the "real" medicine, through psychosomatic effect, or through some hidden pharmaceutical efficacy (e.g., a plant against the dangerous "black water spirits" will be expected to prevent or cure *actual *fevers due to *actual *pathogens in stagnant water).

By contrast, although Ashéninka people do not distinguish "magic" and "real medicinal" uses, they also tend to refer one kind of uses to the other. Nevertheless, they do it in the exactly opposite way. For them, the better conceptual framework to explain the "real" healing power is the working of the "magic" one.

Let us return to the example of snake bites. Like most Amazonian people, the Ashéninka know many herbs, leaves, roots, latexes and barks used to cure them, and actually some of the chemical components could be efficacious against snake venom. When asked about the forms of use, the Ashéninka detail the preparation (rasped or crushed, raw or boiled, etc.), but the very crucial point seems to be so obvious that they usually even forget to mention it: Before any physical use of the medicinal plant, you have to find the snake and kill it, otherwise the medicine is absolutely useless – everyone agrees on this point.

The idea is clear. Killing the snake means destroying the will which yearned for your death. The material medicinal substance is expected to have some physical effect indeed, but first and foremost it must be encompassed in the restoration of the correct relationship. Before this crucial step, the most effective antidote literally does not materialize. It is quite the reverse of our own conceptual hierarchy, whereby the relationship is encompassed in material substances: first my body, the snake venom and the vegetal antidote, and later their interaction.

A knowledgeable Ashéninka knows and uses a very large set of medicinal plants, each of them suited to some specific purpose(s). According to our ethnobotanical surveys, they range from leaves against flu or cicatrizing barks to medicine against rainbow burns, vines for sexual attraction or leaves to improve dogs' hunting ability, and the rate of forest plants used as medicines (in this wide sense) ranges from 76.7 % to 88.6 % [[53] and *TSEMIM *database, see Table 3]. By comparison with such a large and diverse common knowledge, which are then the characteristics of the shaman's skills?

To put it simply, the distinction is quite similar to our own biomedical distinction between symptomatic and aetiological treatments. In most cases I observed or I was told, the illnesses to be treated were exactly the same. The difference is that ordinary people deal with specific, superficial cure, while shamans strike at the root. Nevertheless, both of them are working within the same conceptual framework. As illustrated by the snake bite example, this framework relies on a special stress on relationships, in sharp contrast with our own emphasis on material substances and our basically chemical or mechanical interpretations of diseases and body.

Moreover, the distinction between Ashéninka herbalism and shamanism should be poorly described referring to the classic opposition of body/spirit. Healing is not shared between a bodily dimension reserved for herbal treatments, and a spiritual/soul dimension reserved for shamanic cure. From their own point of view, ordinary people using herbal medicines are acting essentially on the body, but so does the shaman too. Perhaps the latter sounds a little strange, but the reason is that their conception of body and subjectivity is completely different from ours. Bodily materiality and subjective innerness are felt to interact in another way.

#### Ontological background: another conception of the body

This Ashéninka conception is rooted in a peculiar ontological background, the currently well-known "Amerindian perspectivism" [[72-74], and [75] for the specific Ashéninka version]. I briefly summarize the basic points that will be of most interest here. According to this indigenous view, what human people share with all other living beings is not the bodily physical substance, as it is always stressed in Western science (see e.g. phylogeny and ontogenesis, or living cells and biochemistry). What is universally shared is rather a very human-like perception and sociability. Obviously, there are gaps between the different living species, but these gaps are just a matter of specific points of view.

A classic Amazonian illustration should be the peccary/human/jaguar triad. The Ashéninka version is very typical. A white-lipped peccary (*Tayassu pecari*) views its own wandering herd as a foraging human tribe, the swampy hollow where it wallows as a human village, and the wild roots it is eating as domesticated manioc. That is the peccary's sight on its own people, but everything changes when it is looking at outsiders (that is to say, other species). From its prey's standpoint, it views any human hunter in a jaguar form – as well as reciprocally, actual jaguars view their human prey as peccaries, but view other jaguars in a human form, and so forth. Each point of view is directly shaped by the distinct body which defines the peculiar human, animal or vegetal species you belong to.

The overall result is a single but absolutely overcrowded world: There is no real distinction between natural and supernatural, rather "Nature" is divided in a countless (though not infinite) number of disconnected specific points of view. Furthermore, it could be emphasized that these points of view are much more than a simple matter of passive perception. Having a body also means having body-embedded wills. Perhaps it is more conspicuous and understandable in a full-life biodiverse environment like the tropical rainforest, and for hunters who are dealing every day with wild life. Predators or snakes are full of killer mood, prey feels the bodily will to flee or to fight, many other animals and spirits are hungry for certain kinds of human souls (souls as an aspect of the body), some plants are filled with specific psychotropic wills and energy, some others are with healing or poisonous or feeding ones... (about the influence of these beliefs on everyday experience and very basic perceptual processes, see [75].

Human health, hunting, agriculture and general wellbeing largely depend on this complex network of intertwined wills. When something has gone wrong, that is to say when some bad connection ("attacks" and "harmful influences" usually understood to be the sources of illness) or disconnection (mostly in case of hunting or agricultural problems) has harmed human people, the ties and boundaries need to be restored in their favour. That is the shaman's job. He must therefore get directly in touch with the other species, and he cannot do so without dealing with them in their human form. According to the perspectivist thought, the shaman has thus to give up his own human embodiment for a while, and to re-embody in the shape of these other beings, in order to have a temporary access to their specific points of view.

That process of disembodiment and re-embodiment is the basic reason for the typical step-by-step feature of shamanic training. Unlike the herbalist, the shaman does not necessarily have to know and use a wide range of healing plants. He learns them one by one, and it is a slow and long process: first the psychotropic *ayahuasca*, called *kamarámpi *(*Banisteriopsis caapi *(Spruce) Norton, Malpighiaceae) and its main additive *hurúwa *(*Psychotria viridis *R& P., Rubiaceae), then tobacco, according to the Ashéninka hierarchy, later maybe datura (called *sááro*, *Brugmansia candida *Pers., Solanaceae), or more peripheral allies like *thonénto *(*Cavanillesia hylogeiton *Ulbr., Bombacaceae) and *kasáwi *trees (*Duroia hirsuta *(P&E) Benth, Rubiaceae)... Each alliance with a new kind of alien being requires a respective disembodiment/re-embodiment. But the patient building of his personal network of inter-specific ties gives him much more therapeutic efficacy than the herbalist has. It gives him access to a genuine perception of real causes of illnesses, which is crucial for diagnosis, and sometimes for direct healing action. We had no reason to be disappointed by Shoéshi's poor ability to identify medicinal plants. This was just an additional and very peripheral aspect of his job.

### 3. Many forms of healing

The Ashéninka shaman has a wide range of healing techniques at his disposal. This section essentially draws on those I have been told of in details by well-known informants, and most of all, on those I could observe personally. Some of them are close to herbal treatment, others peculiar to shamanic skills. They often echo practices reported in other parts of Amazonia, and present evidences of evolution and transfer. My concern, nevertheless, is not with historical or comparative work. Rather, I want to review them in the light of the Ashéninka constant emphasis on relational aspects of medicine, and of the peculiar body conception I exposed just above. I consider both points as potential keys of understanding of these highly varying practices.

#### Tobacco, thorns and steam baths

In everyday life, an Ashéninka shaman rarely has to resort to his most heroic and accurate healing skills. Just as a Western doctor, the main part of his daily action is to attend to routine cases. I will briefly describe this rather innocuous part of his work first.

The most common healing practice is widespread in the whole Amazonian region or even Amerindian world. The shaman just blows some tobacco smoke -or spits some tobacco juice- on the part of the body of the patient he deems to be the focus of illness. Actually, if we go into particulars, there are many variations among shamans or from one healing session to another: A specific chant might accompany the tobacco blowing or spitting; a thorn or a little piece of charcoal might be sucked out from the patient's body, and presented as the cause of illness; some kind of further medication was often recommended, for instance certain well-known wild plants to be used as leaves baths. But in all cases the distinctive skill of the shaman is always the same. His long frequentation of tobacco and other psychoactive plants gives him an outstanding diagnostic ability. In order to "see" the focus of illness, when faced with ordinary diseases and ailments, he does not need actual disembodiment and re-embodiment any more – besides, a shaman's frequent statement is that he needs to consume less and less hallucinogenic drugs as the years go by.

Another form of everyday healing is steam bathing. In the Ucayali region, it is a specific Ashéninka technique, although Matsigenka women have begun learning it [1], as well as some Yaminahua women from the vicinity, as mentioned before about interethnic borrowing. In fact, steam baths do not belong to the shaman's reserved skills; they are commonly used by specialized Ashéninka women who have some affinity with shamanic talent. What distinguishes the shaman is just a better diagnostic accuracy regarding the selected leaves added to the water and, even more, regarding the divination process after the steam bath.

The patient is placed over a big cooking pot and covered with a large cloth. The pot contains water, leaves of *Pariwana *(*Clarisia biflora *R& P., Moraceae) and sometimes of other plant species, depending on the illness to be healed. Several red-hot axe heads (or stones, in a more traditional version) are progressively put in the water, to produce billows of steam shrouding the patient. After this first step, which lasts about 15 or 20 minutes, the water is thrown away and the leaves are carefully inspected, in order to discover some thorn or piece of bone or charcoal.

Those foreign bodies are understood to be the material support of the illness, rather than its cause. However, the public attention and comments focus exclusively on this material detail, because talking explicitly about the real aetiology, might be problematic. Once again, illness is deemed to be grounded in relationships – that is to say in conscious or unconscious aggressiveness, in this case. Someone sent these thorns or little pieces of charcoal into the patient's body. It could be some harmful beings of the forest ("spirit" or animals), but many times the culprit is deemed to be a relative or a neighbour.

Pointing out the culprit is a real problem for the Ashéninka shaman – and local people. Violence within the ethnic group is strongly prohibited. Unlike the frequent Amazonian pattern of permanent hostility and war between neighbouring local groups, internal feuding has been strongly proscribed for centuries, and shared ethnic identity is always positively stressed in first encounters with unknown visitors. It is an atypical characteristic of all "Campas" (Ashéninka-Asháninka, Matsigenka, Nomatsigenka) and Yanesha-Amuesha: War is only aimed at foreign people [36,13]. In everyday life, the Ashéninka highly value self-control over pain and affects, motions and tone of voice, and interpersonal aggressiveness is rarely allowed to be expressed.

However, conflicts, jealousy and aggressiveness do exist, and are deemed to be an important source of illness. To deal with this uncomfortable contradiction, the shaman has various options at his disposal. As described before, he may engage into individual cures, either with tobacco smoke or with steam baths. He focuses public attention on the thorns or charcoal pieces expelled from the patient's body, and the pointing out of the culprit remains thus as implicit or explicit as he wants – giving him some room for manoeuvre and social management of the problem. The shaman Shoéshi for instance was particularly skilful in this respect.

Additionally, the shaman may engage in another form of agency. To complement this individual or interpersonal side of the cure, he may rely on collective sessions of *ayahuasca*, which are an overall, more preventive healing process- as emphasized by C. Izquierdo about the Matsigenka, health is not an individual, strictly physical and biomedical issue, but also a social, relational one [3].

#### Social healing with ayahuasca

In their current form, the collective sessions of *ayahuasca *are first and foremost a bodily experience. The ceremonial use of the psychotropic mixture was first recorded by Gerald Weiss, who noted that in most cases, "*the ceremony, following a definite if simple format, presents the appearance of a group of people reverently making contact with the good spirits under the leadership of a religious practitioner, even though it is true that they remain passively appreciative spectators of the shaman's virtuosity*." However, "*in one part of Campa territory that *[he]*visited, the ceremony proceeds as described, except that the men take turns singing so that the shaman remains the director of the ceremony but is no longer the only virtuoso*" [7].

Actually, both the form and content of the sessions are evolving. In the early 70's, in the Tambo region, the collective direct participation in drug consumption and songs performance was described by Weiss as an exception. Nowadays, in the Ucayali region, it is a rather common feature. In my opinion, the important point is that the change strengthens the bodily dimension of the experience. Some concrete details will make it clearer.

This kind of *ayahuasca *session is always held at night. Everyone is supposed to participate, though the actual audience is usually restricted to most of the men and some teenagers who want to join them – women stay at home, taking care of the children. All participants sit in a circle in the dark, and each of them goes to the shaman to have a little cup of *ayahuasca*. After waiting for about half an hour, in almost complete silence and immobility (there will be very few motions during the whole session), the hallucinogenic effects of the brew begin to be felt, and the shaman starts the first song. The others follow him, with the same short repetitive song, but everyone sings at his own pace. The voices cover each other, and nobody tries to build a collective song in the sense of trying to reach an acoustic unity.

In fact, everyone is singing for themselves, to celebrate the arrival of the first plants and animals "visiting spirits". In the Ucayali region, the most frequent are the tobacco, *Shéri *or *Pocháro*; the ayahuasca itself (*Banisteriopsis caapi *(Spruce) Norton, Malpighiaceae), addressed as *Hanánero*; the main additive of the brew, *Hurúwa *(*Psychotria viridis *R& P., Rubiaceae); and on the animal side, the Cacique birds *Chówa *and *Tsirótse *(*Psarocolius *sp., *Cacicus cela*, Icteridae). All of them appear in their human form and dressed with their distinctive tunics and ornaments.

Usually, either in regional Spanish or in ethnographic accounts, such human-like apparitions are referred as "spirits" (or "espíritus") of the related plants and animals, visiting the participants in the ritual assembly. However, I assume that such a translation is nothing but a misunderstanding, grounded in our own opposition between steady material bodies and elusive immaterial souls. The Ashéninka are likely to experience it in a quite different way.

In fact, if we pay attention to their genuine wordings (in their own language), the Ashéninka never talk about "visiting spirits", but about the arrival of the specific plant or specific animal itself. It is a question of concepts rather than words. This wording has to be taken all the more seriously since it perfectly fits in with the peculiar ontology of the Ashéninka.

What happens with *ayahuasca *visions then, from an Ashéninka point of view? Under the effect of *ayahuasca*, plants and animals are seen in their human form. It is noteworthy that such a perception is absolutely abnormal, for the Ashéninka as well as for us. In normal circumstances, a hunter *knows *that plants and animals are persons, but this is theoretical knowledge. The Ashéninka are not fanciful people, lost in a sort of blurred irrational fog. As explained before, their animist ontology leads them to pay prime attention to other aspects of living beings' bodies (the will and behavioural trends rather than the morphological outline, the inter-species relationships rather than the material substances), but apart from that, their perception is not *so *different from ours. Plants and animals are perceived as vegetal or animal bodies, which is absolutely consistent with the ontological theory, by the way: Human people have human bodies, so that a man is normally prisoner of his own embodied point of view, just as any other living species is too.

By contrast, when he is faced with plants and animals in human form, the participant in an *ayahuasca *session suddenly becomes able to shift to the perspective of another kind of living beings. According to his ontology, he cannot but understand it as a metamorphosis of his own human body. In fact, it is quite the reverse of the usual Western interpretation as "visiting spirits". What is at stake here is a temporary *bodily *process, whereby a human being assumes the embodied point of view of another species and can meet the *actual *plant or animal. There is no need to appeal to any metaphoric sense here. A literal interpretation of this process of disembodiment/re-embodiment is absolutely consistent with all what an Ashéninka knows and directly *feels *during this experience, in a quite physical sense. One of the initial effects of *ayahuasca *is the sensation of a complete bodily disarticulation, which is probably an inherent effect of the brew (and a rather uncomfortable feeling). It cannot but enhance the conviction of an actual body transformation.

On a social level, such an experience has a great power of healing. Let us imagine now each man from the ceremonial circle, sitting motionless in near absolute dark. For him, the apparitions are a direct and physical experience, much stronger than simple "hallucinogenic visions": He knows and feels that for this exceptional time, his own body is completely reshaped to attend one by one of the visiting plants and animals, and later the solar deity *Páwa*, all of them in their distinctive bright human dress. He is singing at his own pace, firstly for himself and the visitor he personally succeeded in joining, to celebrate the happiness of such an encounter. But at the same time, he hears the voices of all his companions too.

On the one hand, nobody sings in unison. On the other hand, everybody is singing a similar song, that is to say, proves to experience the same happy re-embodiment. The personal experience is also perceived to as a conspicuous general engagement- and let us recall that in this kind of *ayahuasca *sessions, all expected visiting plants, animals and deities are understood to be extremely good and kindly disposed towards all human beings. In this sense, it is a perfect counterpoint for the unacceptable acknowledgement of internal conflicts and tensions. These *ayahuasca *rituals are an affirmation, or even more, a physical experience of collective harmony.

#### Ayahuasca and individual serious illnesses

The collective sessions of *ayahuasca *are the happy side of the disembodiment/re-embodiment processes. There is another, much more dramatic one, also related to *ayahuasca*, but addressing individual patients. When illnesses are particularly serious, common healing techniques like steam baths or tobacco smoke are useless. The patient does not recover, which means that the shaman probably did not identify the main source of illness. After a few days, the shaman understands that the only efficient intervention should be direct work on less obvious aspects of the body.

Such an intervention cannot be explained in biomedical terms, but it clearly relies on a particular conception of diagnosis, as embedded in a plurality of levels and perspectives – as usual, the first step is diagnosis, but levels of diagnosis are various. It is a sort of systemic approach. Illness is understood to be due to some problem within the complex network of intertwined wills that interconnects all living beings, but the point is to circumscribe exactly the problematic relational level. Health depends on the global network, and the harmful influence you discovered at first sight possibly hides another, deeper one.

In these cases of very serious illnesses, the shaman has to consume *ayahuasca*, in order to mobilize his most powerful allies. In the Ucayali region, the first ones were the swallow-tailed kite (*Elanoides forficatus*, Accipitridae) and a tree, called *thonénto *(*Cavanillesia hylogeiton *Ulbr., Bombacaceae). If it did not give the desired effects, he had to appeal then to the help of the most dangerous ones: the giant otter (*Pteronura brasiliensis*, Mustelidae), the king vulture (*Sarcoramphus papa*, Cathartidae), and the jaguar.

It should be noted that these shamanic allies appear in their human form, and act exactly like an Ashéninka human shaman. Their task is to "see" the foreign body – thorn or little piece of charcoal – that makes people ill, and to suck it out from the patient's body, after blowing tobacco smoke on it. The techniques of diagnosis and cure are thus exactly the same as in human world. In fact these allied beings are simply performing usual healing on their own level of reality – or, so to speak, on their own level of "physicality": a jaguar-physicality, a king vulture-physicality, etc. The perspective is absolutely human-like but completely distinct from the human one. What is expected is probably an alternative interpretation of one and the same reality, revealing problems that otherwise remain hidden.

This kind of cure is understood to be extremely dangerous and difficult, and it is strictly reserved to the very shamans. Their vegetal and animal allies belong to the most "powerful" species, which means that the attractive power of their specific perspectives is particularly strong.

#### The dangers of disembodiment

Temporary disembodiment is a well-known Amerindian topic [72,73,76]. It is dangerous because you may be kept in another being's perspective, without being able to come back to the human one. It may occur while dreaming, walking absent-minded in the forest or giving into the appeal of recently dead relatives, and it is one of the greatest fears an Ashéninka may feel. Firstly your human body appears to be unchanged but it is gradually affected, and finally you die: Death means being caught in the sight of the other.

Therefore, in Ashéninka thought, death always comes from outside, but "outside" and "inside" are relative concepts: The boundaries are insecure and unstable, they may be permanently crossed over or twisted by the wills of other beings. That is because the world is not "out there", unfolding around human people. As any other living species, we are embedded in it as direct partakers of its networks. It is a dense and overcrowded world, but within this world, despite its wideness, energies and material substances are finite (a crucial point of Amazonian, and probably Amerindian thought, which I think we never stress enough – see [77,78]). Due to this limitation, each species and being has to feed on one another in order to live and grow.

Indigenous theories about generalized predation are another crucial Amazonian topic [77-81]. At first sight, they look a little bit like our own ecological or Darwinian approaches, but in order to avoid any misunderstanding we have to point out a crucial difference. From an Ashéninka point of view, what happens is not exactly a struggle for life between species and/or individuals, understood as separated entities. Once again, everything remains grounded in relationships. Actually, the other side of predation is often seduction, which can induce a *consenting *bodily metamorphosis and assimilation to the other.

An illustration of this aspect is the case of the *peyári*. The word could be translated as "ghost" or "ghost soul", though the concepts about "souls" are rather distinct from ours. The *peyári *arises when people die. It is the part of a recent deceased which stays there for a time, often embodied in a partial or complete animal shape (a man with a sloth head, or a deer with a strange behaviour, for instance), whereas another kind of soul simply goes away (as usual in Amazonia, souls are several, and may undergo quite different fates).

The Ashéninka are usually reluctant to express fear and pain, and in everyday life the presence of a *peyári *is the only situation which repeatedly provokes real panic. It is quite understandable. For some weeks or months after death, the *peyári *remains walking around the village, especially at night-time, and calls to its former friends and relatives to take them along. It is understood as a frequent cause of illness and death. In this sense, the *peyári *is a predator and a killer. However, from its own point of view the situation is completely different. The reason for its harmful behaviour is nothing but sorrow and loneliness; it simply does not want to leave its village and its family. In fact, it just wishes some company before going away – and of course it therefore tries to convince its closest and most loved friends and relatives. For the remaining living people, there is thus a great temptation to pay attention to this sorrow. It comes from their recently dead wife, brother or close friend. But if they give in to this appeal, they fall in the *peyári *point of view, which entails nothing but illness and death, from a human point of view.

Local strategies to resist the dangerous attraction of death people vary throughout Amazonia [41,82-84], but they are usually characterized by the same kind of emotional ambiguity as among the Ashéninka. Regarding ethnomedical concepts, another noteworthy point is the ontological status of the *peyári*. I mentioned above examples of its sloth head or deer body. Just like its Araweté equivalent, *ta'äwé *[85], this curious "soul" is not conceived of without a specific embodiment (I mean "specific" in the sense of belonging to a particular species of beings, with distinctive physical and behavioural characteristics). Rather than against a "supernatural spirit", the dead people's relatives have to fight against the cannibalistic seduction of a quite material being. Once again, the struggle is about the body: The *peyári*'s aim is to reshape living people according to its own bodily perspective.

There are so a lot of risks and temptations in the "disintegration" of the Self. For the Ashéninka, the boundaries of the Self and the body are fragile and unstable, and staying alive as a human being means firstly a permanent fight against disintegration. Many other living beings are waiting for it, with very few exceptions: your kin, who share your same food, some allied animal and vegetal species, and perhaps your whole ethnic group, since unlike many other Amazonian peoples, the Ashéninka established a permanent truce between themselves, prohibiting physical warfare and shamanic aggression. With all the others, predation is the mainspring of life, of growth, health and illness, and of death. All preys and predators (including the "spirits") are individual, material beings, without real distinction between natural and supernatural worlds – or natural and supernatural diseases: The points of view are distinct indeed, but this is just a matter of distinctive bodies. This theory of general predation and bodies' discontinuity probably gives the overall framework of what healing means among the Ashéninka.

## Conclusion

The practices I have presented are manifold. They include herbal interventions, which could be, deceptively, compared to our own approach of medicinal substances, and the highly varying forms of healing performed by the shamans. As a conclusion, I shall synthesize my results, compare the shamanic and the herbalist approaches and question eventually their coexistence and hierarchy.

Herbalism and shamanism do not address two different kinds of illnesses, according to countless personal observations. In other parts of Amazonia, a sharing of "natural diseases" (reserved to herbal treatment) and "supernatural diseases" (reserved to shamanic cure) has been discussed, as well as a partial overlapping between both categories [71]. Among the Ashéninka, such distinction does not make sense. The illnesses are essentially the same. What differs are the forms of treatment and their degrees of efficiency. The choice depends on the seriousness of the disease, but both herbalism and shamanism address similar issues and are embedded in one and the same rationality. This peculiar rationality was precisely my central concern. The successive steps of the analysis could be summarized as following.

First, the analysis of the regional interethnic borrowing system has demonstrated the crucial opposition between material substances and interpersonal relationships. I have shown that Western scientists and indigenous stakeholders do not apply the same criteria to assess ethnobotanical knowledge. For the former, a medicinal plant is an object, and the focus is on its objective, material effect. For the latter, a plant is a being, and its therapeutic efficiency is expected from its special relationship with definite human groups.

In the 2^nd ^section ("Who is a good healer?"), the relational dimension has appeared to be a priority in all fields of Ashéninka ethnomedicine. It involves relationships with animal and plant species as well as human beings. It defines the superior healer's status of the shaman, as a specialist in relationships with other beings, and is the reason why a shaman does not necessarily need to know a wide range of medicinal plants. Even herbal treatment, which at first sight relies on material effects of plant substances, is embedded in an underlying relational dimension, deemed to be critical for its actual efficiency.

Material effects are embedded in relationships. However, I have also shown that it does not mean some priority of a "spirit" or "soul" dimension. Even the shaman is deemed to act directly on the body, since the Ashéninka conceive the link between bodily materiality and subjective innerness quite differently from Western thought.

I have illustrated this point in the 3^rd ^section, by reviewing the many forms of healing I met and was told about. This review outlines a world of general predation. All species of beings feed on each other (all of them have a body, including the "spirits" and "souls"), which is the mainspring of life, of growth, health and illness, and of death.

### Predation, body nurturing and body shifting

Both Ashéninka herbalism and shamanism perfectly fit in with these views. Rather than two specialized, separate fields of healing, they form two distinct *expressions *of the same issues. General predation in a world of limited resources of substances and energy may indeed be expressed in two ways.

The first one is through shamanic language of disembodiment/re-embodiment. Predation (or illness: you are ill because another kind of living being is feeding on you) is then a sudden shift of your body towards the perspective (or the shape: it is exactly the same indeed) of this harmful other being. If you are not a shaman, you cannot see it immediately – and that is why the shaman's knowledge is a superior one: His ability for controlled temporary disembodiment gives him outstanding skills in diagnosing, and also in healing the more severe cases, when the only effective intervention is direct work on the overall shape of ill people.

By contrast, if you are just an ordinary Ashéninka person, you do not have access to such a vision and aetiological treatment, but you see the problem from outside, as it is expressed in the second healing (or body) language, namely the symptomatic one. Ill people become progressively sick and thin, substances are going slowly out of their body (another kind of living being is feeding on them: it is exactly the same). It is a view from outside, of course, but you also know about the other theory. If the symptoms are obvious, if the ailment is limited, and if you are a good herbalist, you can guess what is happening, and go to the forest in search of the suitable medicinal plants. Maybe they will be strong enough to fight against the harmful predator – it is a process you will see from your outer symptomatic point of view, as an addition of vegetal substance counterbalancing the loss induced by the illness.

This second expression of health, illness and body dynamics is obviously less thoroughgoing than the first one (at least among the Ashéninka), but usually it is sufficient to satisfy the needs of everyday life and use. When a snake bites you, the reason of the problem is obvious enough: as mentioned, you pay your debt to the perspectivist theory killing the snake, and then you look for the suitable medicinal plants. When you want to make preventive medicine or to cure little ailments of your children, you give them leaf baths: you know perfectly that these kinds of leaves are against specific cannibal beings ("spirits") of the forest, but you do not need to access their peculiar point of view, you are just "strengthening the body" of your children – and so forth. The shaman uses his personal skills in perceiving foreign bodily perspectives to intervene directly in the overall network of all living beings, involving men, plants, animals and spirits. Ordinary people acknowledge the same conceptual framework but confine their therapeutic action to a mere symptomatic level.

Nevertheless, there is a problem of coexistence and hierarchy. Shamanic knowledge is deemed to be much more thoroughgoing, but in fact, the ordinary people's symptomatic approach, closely related to the concept of "body nurturing" [86], works as an alternative folk theory. Among the Ashéninka, it might appear as consistent as the shamanic approach. It refers to the idea of a continuous construction of the body that we also find in the many dietary prescriptions and proscriptions. Every kind of living being feeds on some other ones, but has to do so very carefully. Substances are circulating from one being to another, but that is precisely why they are not just inert stuff – in whole Amazonia, it is particularly conspicuous in the couvade rituals [87,88]. There are risks of unbalance or contamination, especially on the most sensitive moments of change: birth and infant's growth, convalescence, shamanic training...

Among the Ashéninka, nevertheless, this theory of continuous body nurturing is less emphasized than the other one, namely perspectivism as a theory of sudden body shifting. Dietary prescriptions and proscriptions are often partially disregarded. The stronger prohibitions are related to behaviour rather than food, and to alien perspectives rather than powerful substances. As an example, the Ashéninka avoid very carefully *looking *at some animal species deemed to be *mirítse*, literally "orphan", like the anaconda or the giant anteater: if you do so, somebody of your close kin will die (you fell in this orphan's point of view). Even in the case of recent birth, which in other parts is the most typical moment for body nurturing prohibitions, the main danger is with perspective shifting as much as with substances contamination. If the parents walk in the forest without protecting themselves (by spitting the juice of a specific "piri-piri" bulb on their own feet), some animal species, especially no-venomous little snakes called *torótse *and *shonkishonkítse *(n.i.), will smell their trail (without touching their feet), so that later the newborn's "soul" follows them.

Both body nurturing and body shifting are crucial topics in Amazonia. They entail varying indigenous statements and practices, but both seem to be found side by side everywhere in the region. At the moment, I suspect that they are working as two competing integrative conceptual/perceptual schemata, neither really opposite nor really complementary (the first one refers to temporal continuity, the second one to species discontinuity). Among the Ashéninka, body nurturing is encompassed by the body shifting model, but the balance between both seems to be quite different amongst each ethnic group. For instance, by sharp contrast with the Ashéninka, some of their neighbours seem to stress much more on the other explanatory model: the body nurturing, as a circulation of substances among the Kulina [4], or as an accumulation of embodied knowledge among the Cashinahua [5].

Perhaps a good question to ask would be which model encompasses the other in each case. As suggested by the Ashéninka example, essential criteria for assessing this issue should be the trend to emphasize one kind or other of aetiological explanations, and (on the other side of the coin) the trend to respect more carefully one kind or other of dietary or behavioural prescriptions and prohibitions. Beyond the Ashéninka specific case, the point probably calls for further research.

## Declaration of competing interests

The author(s) declare that they have no competing interests.
